# The Genetic Spectrum of Familial Hypertriglyceridemia in Oman

**DOI:** 10.3389/fgene.2022.886182

**Published:** 2022-05-20

**Authors:** Khalid Al-Waili, Khalid Al-Rasadi, Muna Al-Bulushi, Mohammed Habais, Abdullah Al-Mujaini, Saif Al-Yaarubi, Antoine Rimbert, Razan Zadjali, Pegah Moradi Khaniabadi, Hamida Al-Barwani, Sana Hasary, Zayana M. Al-Dahmani, Hala Al-Badi, Almundher Al-Maawali, Fahad Zadjali

**Affiliations:** ^1^ Department of Clinical Biochemistry, College of Medicine and Health Sciences, Sultan Qaboos University, Muscat, Oman; ^2^ Medical Research Centre, College of Medicine and Health Sciences, Department of Biochemistry, Sultan Qaboos University, Muscat, Oman; ^3^ Department of Ophthalmology, College of Medicine and Health Sciences, Sultan Qaboos University, Muscat, Oman; ^4^ Department of Child Health, Sultan Qaboos University Hospital, Sultan Qaboos University, Muscat, Oman; ^5^ Nantes Université, CHU Nantes, CNRS, INSERM, L’institut du Thorax, Nantes, France; ^6^ Sultan Qaboos Comprehensive Cancer Center, Muscat, Oman; ^7^ Department of Genetics, College of Medicine and Health Sciences, Sultan Qaboos University, Muscat, Oman

**Keywords:** familial hypertriglyceridemia, lipoprotein lipase, gene variant, gene mutation, LPL

## Abstract

Familial hypertriglyceridemia (F-HTG) is an autosomal disorder that causes severe elevation of serum triglyceride levels. It is caused by genetic alterations in *LPL*, *APOC2*, *APOA5*, *LMF1*, and *GPIHBP1* genes. The mutation spectrum of F-HTG in Arabic populations is limited. Here, we report the genetic spectrum of six families of F-HTG of Arab ancestry in Oman. Methods: six Omani families affected with triglyceride levels >11.2 mmol/L were included in this study. Ampli-Seq sequencing of the selected gene panels was performed. Whole-exome sequencing and copy number variant analysis were also performed in cases with negative exome results. Three novel pathogenic missense variants in the *LPL* gene were identified, p.M328T, p.H229L, and p.S286G, along with a novel splice variant c.1322+15T > G. The *LPL* p.H229L variant existed in double heterozygous mutation with the *APOA5* gene p.V153M variant. One family had a homozygous mutation in the *LMF1* gene (c.G107A; p.G36D) and a heterozygous mutation in the *LPL* gene (c.G106A; p.D36N). All affected subjects did not have a serum deficiency of LPL protein. Genetic analysis in one family did not show any pathogenic variants even after whole-exome sequencing. These novel *LPL* and *APOA5* mutations are not reported in other ethnic groups. This suggests that patients with F-HTG in Oman have a founder effect and are genetically unique. This warrants further analysis of patients of F-HTG in the Middle East for preventative and counseling purposes to limit the spread of the disease in a population of high consanguinity.

## Introduction

Hypertriglyceridemia (HTG) is a metabolic disorder characterized by elevated levels of fasting serum triglyceride (TG) (Lewis et al., 2015; Lahoz and Mostaza, 2021). Based on the Endocrine Society Clinical Practice Guideline, HTG is classified as mild (TG 1.7–2.2 mmol/L), moderate (TG 2.3–11.2 mmol/L), severe (TG 11.3–22.5 mmol/L), and very severe (TG ≥ 22.6 mmol/L) (Berglund et al., 2012). Familial hypertriglyceridemia (F-HTG) is a rare genetic disorder that is found in less than 5% of HTG cases. It is caused by mutations in at least five different genes, namely, lipoprotein lipase (*LPL*), apolipoprotein C-II (*APOC2*), apolipoprotein A-5 (*APOA5*), glycosylphosphatidylinositol-anchored high-density lipoprotein-binding protein (*GPIHBP1*), and lipase maturation factor 1 (*LMF1*) genes. Secondary HTGs are more frequent and attributed to obesity-related diabetes mellitus, hypothyroidism, nephrotic syndrome, or drug-induced disorders (Clement et al., 2014; Cruz-Bautista et al., 2021).

The most severe forms of HTG are seen in both familial chylomicronemia (type I hyperlipidemia, OMIM#238600) and primary mixed hyperlipidemia (type V hyperlipidemia, OMIM#144650). Type I hyperlipidemia is characterized by defective clearance of chylomicrons and has an isolated elevation of blood chylomicrons, while type V hyperlipidemia shows elevation in both chylomicrons and very-low-density lipoprotein (VLDL) (Hegele, 2009; Brandts and Ray, 2021). The LPL enzyme is expressed on the surface of endothelial cells of capillaries, and it releases fatty acids from triglycerides from the intestine and liver into the bloodstream. A higher density of disease-causing mutation mainly exists in the middle of the *LPL* gene. The majority of loss-of-function mutations are located in exons 5 and 6 of the *LPL* gene since these exons encode important functional domains of the enzyme (Murthy et al., 1996; Shakhtshneider et al., 2021; Wu et al., 2021). Up to date, there is no study conducted on common genes associated with F-HTG in the Arabian region, with the exception of a case report from Israel on an Arab decent child with a mutation in the *LPL* gene (p.Arg270His) and p.Ser286Arg in a Moroccan patient (Bouabdellah et al., 2015; Dron and Hegele, 2017). In this study, we identified six families of F-HTG of Arab ancestry and reported their genetic causes. We further investigated the genetic causes of F-HTG and correlated the genotypes with LPL protein expression.

## Materials and Methods

### Study Subjects

Blood samples were collected from patients with a clinical diagnosis of F-HTG at the lipid clinic in Sultan Qaboos University Hospital, Oman. Samples were also collected from their relatives. The study involved six families resulting in a cohort of 28 individuals consisting of 12 F-HTG affected patients (11 males and 1 female). Patients with mild or moderate HTG, history of uncontrolled diabetes, hypothyroidism, significant proteinuria or nephrotic syndrome, obesity, alcohol consumption, and paraproteinemia disorders were excluded from the study. Blood samples were collected after overnight fasting from all individuals for molecular and functional studies. Serum samples were collected and stored at −80°C for further analysis. The aliquoted serum samples were used for lipoprotein lipase immunoblotting. The whole blood EDTA samples were stored immediately at −80°C for DNA extraction. Family pedigrees were illustrated using cranefoot_3.2.3 software (Mäkinen et al., 2005).

### Genetic Analysis and Bioinformatics

Genomic DNAs were extracted from whole blood using Qiagen mini kit (QIAamp DNA Mini). Ampli-Seq technology on the Ion Proton platform (Thermo Fisher Scientific, Inc.) was performed to sequence the F-HTG gene panel: *LPL*, *APOC2*, *APOA5*, *GPIHBP1*, and *LMF1*. The exon flanking introns and promotor regions were included in the panel. For individuals with negative exome results, DNA samples were sent to a service provider for whole-exome sequencing, Macrogen © South Korea. DNA was barcoded and enriched for the coding exons of targeted genes using hybrid capture technology (Agilent SureSelect Human All-exons-V6). Prepared DNA libraries were then sequenced using Next-Generation Sequencing (NGS) technology (NovaSeq 6000, 150) bp paired-end, at 200X coverage).

Sequencing data were processed by the Torrent Suite and reads were aligned to the hg19 reference genome. Variant call files were then generated using Torrent Variant Caller plugins. Variant annotation was performed using ANNOVAR and variants were linked to ExAC, and Greater Middle East-Arabian Peninsula (GME-AP) databases for allele frequencies. . Effect of amino acid changes was predicted by LRT, CADD, and MutationTaster. Pathogenic variants were identified from allele frequency of <1% or novel, coverage depth >30, the damaging effect from at least two of the three prediction algorithms, and segregation with the disease in the family. For CNV analysis, we used the CNVkit tool for patients with no pathogenic variant identified in the LPL gene (Talevich et al., 2016).

### Immunoblotting

The serum of all affected cases including two serum samples from the control group was examined. Clear serum was collected from the bottom of the tube (milky ring avoided) after maximum centrifugation. A total of 200 μl serum was first cleared from IgG using washed magnetic beads (PureProteome™ Protein A/G Mix Magnetic Beads, Millipore). 1 μl of LPL antibody (LPL Antibody F-1, mouse monoclonal IgG, Santa Cruz Biotechnology) was added to IgG-cleared serum and incubated overnight in the cold room. Then LPL protein was immunoprecipitated using washed IgG beads under rotation for 3 h at room temperature. Then final beads were washed and mixed with reducing buffer. Proteins were separated in 12% Sodium dodecyl sulfate-polyacrylamide gel for electrophoresis. Western blot was performed using primary LPL antibody. We used total protein loading as a normalization technique (Aldridge et al., 2008). Total lane intensity was used to measure total protein loading per sample.

## Results

### Family Pedigrees

**FIGURE 1:**
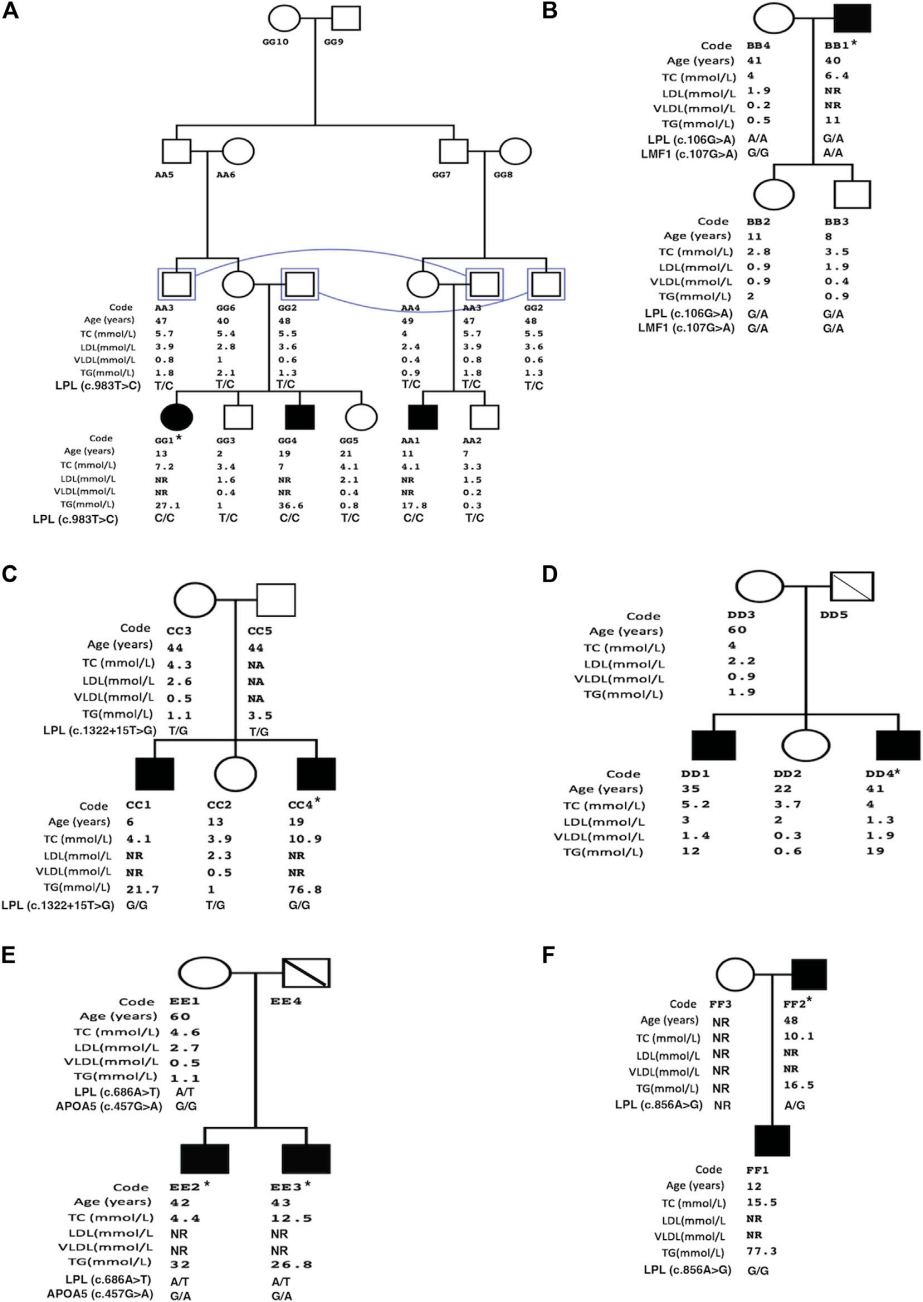
Family pedigree structures of patients with familial hypertriglyceridemia. Illustrates family trees of study groups (A= Family1 to F= Family 6). Study group data includes age, total cholesterol (TC), low-density lipoprotein (LDL), very-low-density lipoprotein (VLDL), and triglycerides (TG). Squares and circles represent male and female family members, respectively, and filled symbols represent affected subjects with familial hypertriglyceridemia. * Index case, NR: not recorded, connecting line: same individual, diagonal line: deceased subject.

The lipid profiles of the affected family members are described in Figure 1. Table 1 summarizes the identified mutations in the patients.

**TABLE 1:** List of pathogenic variants in the families.

Gene	APOA5	LMF1	LPL				
cDNA change	c.457G > A	c.107G > A	c.983T > C	c.106G > A	c.1322+15T > G	c.686A > T	c.856A > G
A.A change	p.V153M	p.G36D	p.M328T	p.D36N	-	p.H229L	p.S286G
exon No.	5	1	6	2	8	5	6
ExAC-All	0.02	0.18	Novel	0.015	Novel	Novel	Novel
GME-AP	0.06	0.06	Novel	0.018	Novel	Novel	Novel
Mutation Taster prediction	Polymorphism		Damaging	Probably harmless	-	Damaging	Damaging
LRT prediction	Neutral	Neutral	Damaging	Neutral	-	Damaging	Damaging
CADD	1.57	1.03	17.51	10.57	-	29.3	16.92

Allele frequencies were reported using ExAC-all and GME-AP databases. Exonic variant effects were assessed using the Mutation Taster, LRT, and CADD prediction tool.

Family 1 (Figure 1A) is made of double cousin marriage with three affected individuals AA1 (age 11-yrs), GG1 (12-yrs), and GG4 (19yrs) with a severe elevation of triglycerides. Clinically, three patients suffered from recurrent pancreatitis and did not have lipemia retinalis, hepatosteatosis, and a history of cardiovascular disease, obesity, alcoholism, renal disease, or diabetes mellitus. Sequencing of the 5 candidate genes showed a novel *LPL* gene variant (c.983T > C, p.M328T) with all affected siblings being homozygous. The rest of the family members were found heterozygous carriers. The *LPL* p.M328T mutation is not reported in the *LPL*-Leiden Open Variation Database [14] and is located in exon 6 of the gene with the predicted damaging effects, Table 1.

In family 2, only one affected member (father, BB1) had clinical criteria for F-HTG with severe triglyceride levels. The father had obesity with no signs of lipemia retinalis. There was no history of acute pancreatitis, diabetes mellitus, renal disease, cardiovascular disease, or steatohepatitis in the family. The analysis of both targeted and whole-exome sequencing data did not identify pathogenic variants. The condition in the father could be due to double alteration in *LPL* (c.G106A; p.D36N) and *LMF1* (c.G107A; p.G36D). The *LPL* variant p.D36N is likely a benign variant (rs1801177) shown by the prediction algorithms, Table 1. The family’s two siblings were heterozygous in two genes variants, however, they showed normal triglyceride levels. Yet, the father was homozygous for the *LMF1* (NM_001352020) p.G36D variant which may explain his severe phenotype. Furthermore, analysis of whole-exome sequencing data did not identify any CNV nor pathogenic variants in glycerol-3-phosphate dehydrogenase 1 (*GPD1*) to exclude possible Transient Infantile Hypertriglyceridemia.

In Family 3, two of the five members were affected with severe HTG. The affected sons, CC1(6 yrs) and CC4 (19 yrs) had fasting TG levels of 21.7 mmol/L and 76.8 mmol/L, respectively. Recurrent pancreatitis was present in the older son only. Both affected siblings did not have signs of lipemia retinalis and had normal blood glucose levels. Genetic analysis of family 3 showed a splice variant in the exon8-intron8 junction of the *LPL* gene. Both affected sons were homozygous, while the unaffected sibling and parents were heterozygous carriers.

In family 4, fasting TG levels of the affected males DD1 (35 yrs) and DD4 (41 yrs) were severely high at 12 mmol/L and 19 mmol/L, respectively. TG level of the mother (DD3) was normal. There was no history of acute pancreatitis and signs of lipemia retinalis in the family. Patient DD1 had obesity and steatohepatitis associated with hepatitis B infection. Patient DD4 had a history of transient ischemic heart disease and stage 2 chronic kidney disease. Using filtering strategy, no CNV nor pathogenic variants were identified in the Ampli-Seq panel or whole-exome sequencing.

Family 5 consisted of two affected patients with severe TG levels and a mother with normal TG levels (Figure 1). Recurrent acute pancreatitis was present in the affected patients but not in the mother. Genetic analysis of the two affected patients revealed a novel pathogenic *LPL* gene variant (c.686A > T, p.H229L) according to the predictions algorithm. However, the healthy mother was also heterozygous like the affected siblings. Therefore, we analyzed the family using a double heterozygous model. We identified a polymorphic variant in the *APOA5* gene in affected patients but not in the mother. Therefore, this family had a compound heterozygous mutation in *LPL* and *APOA5* genes.

In family 6, the TG levels of an affected son (FF1) was 77.3 mmol/L which was higher than the affected father (FF2) with a TG level of 16.5 mmol/L. Both patients had recurrent attacks of acute pancreatitis and no sign of lipemia retinalis. The affected father had a history of diabetes mellitus with nephropathy. There was no history of steatohepatitis and cardiovascular disease in the family. A novel *LPL* gene variation (c.856A > G, p.S286G) was identified in the existing panel screening. The affected son with severely elevated TG was homozygous at the mutation while the father with a lower TG level was heterozygous (Figure 1). The mother did not attend the clinic for investigation.

### Serum LPL Protein Levels

**FIGURE 2:**
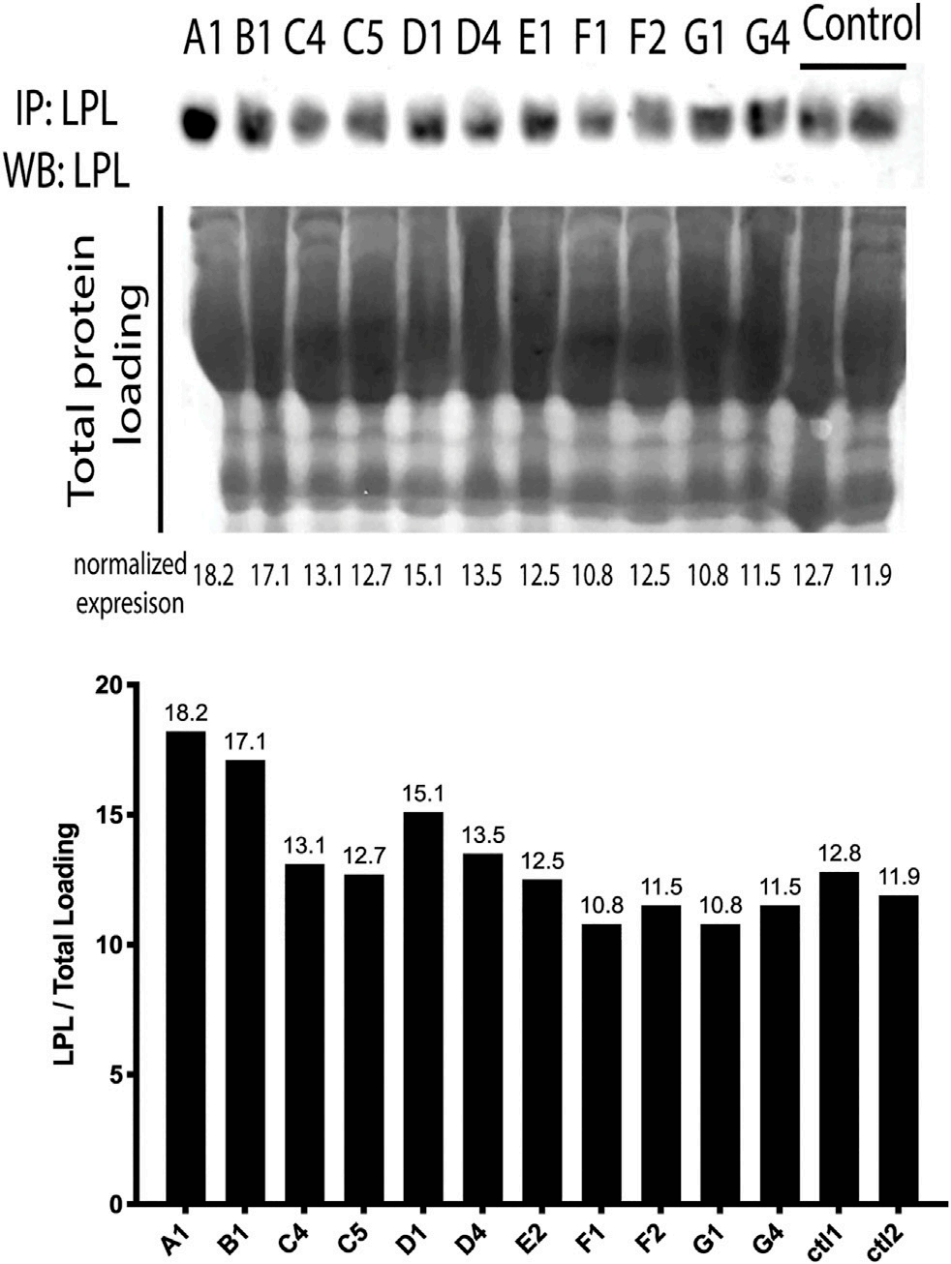
LPL protein levels in the serum of affected individuals. The LPL densities were normalized to total protein loading. Both Ctl1 and Ctl2 are normal control samples. Individuals ‘labels are as follows: A1:AA1, B1:BB1, C4:CC4, C5:CC5, D1:DD1, D4:DD4, E2: EE2, F1: FF1, F2: FF2, G1: GG1, G4: GG4.

We then analyzed the LPL protein expression in serum to investigate if patients had an absolute deficiency of enzyme. To normalize the LPL expression, we used total protein loading in each Western blot lane, Figure 2. LPL was immunoprecipitated and expression was compared to two control cases. We found that LPL protein was expressed in all cases of F-HTG, which indicates that mutations impact the protein’s functioning rather than its synthesis or stability.

## Discussion

Primary disorders of triglyceride metabolism result from genetic defects in triglyceride synthesis and metabolism. Here, we report the genetic spectrum of six families with severe familial hypertriglyceridemia of Arab ancestry in Oman. Three families presented with novel pathogenic variants in the *LPL* gene: two missense variants p.M328T and p.S286G and one splice variant c.1322+15T > G. One family had double heterozygous variants in *LPL* (p.H229L) and *APOA5* gene (p.V153M). Another family had double heterozygosity between *LPL* (p.G36N) and homozygous *LMF1* mutation (p.G36D). All affected subjects did not have serum deficiency of LPL protein level and most likely the variants affect the lipolytic activity of LPL. Previous studies on other disease-causing mutations also the variable effect of protein levels of LPL (Kozaki et al., 1993; Hooper et al., 2008). Genetic analysis in one family did not show any pathogenic variants even with whole-exome sequencing.

The novel pathogenic missense variant in the *LPL* gene (rs1181582051, c.983T > C, p.M328T) causes a substitution in the carboxy-terminal structural region of LPL, which spans 313–448 amino acids, on the highly conserved beta-strand (uniport: LIPL_HUMAN). The LPL-mediated lipoprotein absorption through the cell surface receptor catalyzes the first contact between LPL and the substrate in this domain (Murthy et al., 1996). Furthermore, beta-strands are crucial secondary structural elements that affect protein folding, structure, and topology, as well as the protein’s functional confirmation and activity (Henderson et al., 1993; Jin et al., 2021). When compared to controls, this variant was detected in family 1 members who had normal LPL expression. This indicates that the mutation had no effect on the stability of the protein.

The novel missense mutation p.S286G in exon 6 of the *LPL* gene occurs in a less-conserved region of the gene. The 286 residues are located very close to the S-S bridge between the two cysteine residues (278–283) which plays an important role in stabilizing the heparin-binding (R279-K280 and R282) (Murthy et al., 1996). A previous case with F-HTG was reported in a Moroccan patient but with homozygous c.858T > A/p.S286R mutation (Bouabdellah et al., 2015). The heterozygous p.S286G carriers in the study family had triglycerides half levels of those of homozygote cases. suggesting that the mutation has a dose effect.

A missense mutation (c.686A > T, p.H229L) in the *LPL* gene has been detected in a family of 4 members even in the mother with normal TG levels. To the best of our knowledge, this mutation is not reported in any population. Amino acid residues from 228 to 234 of LPL make up the ß-strand in the N -terminal domain, UniProt. Studies by Dugi et al. (1992) and Henderson et al. (1993) highlighted the region from Cys218 to Cys 239 as the lid/loop domain which is stabilized by one of the four disulfide bonds of LPL protein. The lid/loop is involved in the hydrolysis of some triglycerides and phospholipids, the initial identification of the lipase substrate, and giving excess to the catalytic triad (Goldberg and Chait, 2020). At the lid proximal and distal regions, the charge and periodicity play a critical role in maintaining the catalytic activity and the apical residues of the loop contribute minimally to LPL function (Dron and Hegele, 2017). Therefore, to have a dominant effect on serum TG levels, the patient phenotype suggested the presence of a compound heterozygous model. Earlier reports show that more than two-thirds of F-HTG is caused by an autosomal recessive variant in the *LPL* gene [22, 23]. Less abundant cases are due to autosomal recessive or double heterozygous *LPL* variants with variants in *LMF1*, *APOA5*, *APOC2,* or *GPIHBP1* gene (Basel-Vanagaite et al., 2012; Goldberg and Chait, 2020). The double heterozygosity affects the function and maturity of LPL leading to elevated serum TG. LPL activity requires apoC2 and apoA5, while the maturity and transport of LPL are controlled by LMF1. GPIHBP1 is required for surface attachment of LPL at the endothelial cell surface. The compound heterozygosity in family B explains that the presence of homozygous p.G36D variant in *LMF1* along with heterozygous changes in *LPL* and *GPIHBP1* causes the severe elevation in the affected father BB1. The p.G36D of *LMF1* was earlier reported in a Thai family with F-HTG with one patient being homozygous and 4 were heterozygous (Plengpanich et al., 2020).

None of the cases presented clinically as transient infantile hypertriglyceridemia. These patients usually present with moderate hypertriglyceridemia at birth and gradually decrease with growth [24]. The condition is caused by an autosomal recessive mutation in the glycerol-3-phosphate dehydrogenase 1 gene (*GPD1*).

## Conclusion

Hypertriglyceridemia can present as a monogenic (familial) or polygenic disease. Monogenic hypertriglyceridemia conditions are very rare and commonly cause severe elevation of serum TG. Here, we described six families of F-HTG in Oman. Four pathogenic variants in the *LPL* gene were identified: p.M328T, c.1322+15T > G. p.S286G, and p.H229L. The latter variant existed as double heterozygous with the *APOA5* variant p.V153M variant. One family with severe hypertriglyceridemia resulted in negative exome data. Further whole-genome sequencing and epigenetic studies are needed to explore the genetic basis in these families. The reported novel mutations in the study suggest that patients with F-HTG in Oman may have a founder effect and are genetically unique. This warrants further analysis of patients with F-HTG in the Middle East for the preventative and counseling purpose to limit the spread of the disease in a population of high consanguinity.

## Data Availability

The datasets for novel mutation presented in this study can be found in online repositories: Global Variome shared LOVD (https://databases.lovd.nl/shared/individuals/LPL) with accession individual I.D numbers 00408363, 00408402, 00408403, 00408404.
